# Dynamics of Porcine Circovirus Type 3 Detection in Pre-Weaning Piglets: Insight From Multiple Sampling Methods

**DOI:** 10.1155/tbed/4735187

**Published:** 2025-01-24

**Authors:** Danchen Aaron Yang, Meng Li, Yi Wang, Kangning Zhao, Qiyang Zhang, Richard Anthony Laven, Zhen Yang

**Affiliations:** ^1^College of Veterinary Medicine, Nanjing Agricultural University, Nanjing 210095, China; ^2^Huanshan Group Co., Ltd., Qingdao 266061, China; ^3^School of Veterinary Science, Massey University, Palmerston North 4442, New Zealand

**Keywords:** dynamics, piglets, porcine circovirus type 3, sow farm, virus detection

## Abstract

Porcine circovirus type 3 (PCV3) has been identified worldwide and is associated with reproductive and systemic diseases, yet the dynamics of PCV3 within pig farms remain unclear. Building upon our previous study, which initialised comparisons of different sample types for the detection of PCV3 in a sow farm, this study expanded both the range of sample types and the timeline of sampling in piglets and sows to better understand the PCV3 dynamics. This study collected two additional sample types—oropharyngeal swab (OS) and oral fluid (OF) along with placental umbilical cord (PUC) blood and processing fluid (PF) that were used in the previous study. Data were collected from July to August and October 2022; the aforementioned four sample types from 51 litters were collected, and additional OS samples were collected from two to three identified piglets per litter on days 1, 7, 14, and 21 post-farrowing. Besides, blood swabs were taken from 135 sows subject to both PCR test and oestrogen measurement. PF showed the highest detection rates (50/51), while OS and OF revealed 33/51 (95% confidence interval [CI]: 51.2%–76.8%) and 37/51 (95% CI: 59.5%–83.5%) detection rates; both were higher than that of PUC blood (22/51, 95% CI: 30.2%–56.8%). Despite the similarity between OS and OF samples, they did not identify the same population as infected, as the agreement between the samples was only fair at 90% level. The Bayesian generalised linear mixed model suggested PCV3 was more likely to be detected in both OS and OF compared to PUC blood, and PCV3 was present in the farrowing room throughout the pre-weaning period using an OS. Finally, we observed higher PCV3 detection rates in sows after farrowing; however, no evidence was found that such a pattern was associated with the decreased concentration of oestrogen.

## 1. Introduction

Porcine circovirus type 3 (PCV3), a novel single-stranded circular DNA virus, was first identified in 2015 through metagenomic sequencing in sows with poor reproductive performance, high miscarriage rates, and increased mortality on pig farms in North Carolina, USA [1]. To date, PCV3 infections have been reported across most of the major pork-producing countries in the world [2–4]. Although the majority of PCV3 infections are subclinical and mild, research over the past decade has identified two major clinical presentations associated with PCV-3 infection: (a) reproductive disease that affects sows and fetuses, and (b) systemic disease that affects piglets before and after weaning [5].

PCV3 can be found in multiple organs of infected pigs and can be shed via a wide range of routes, including faeces, body fluids, and colostrum [6–8]. There are thus multiple samples that can be taken when trying to estimate PCV3 prevalence within a herd [9]. However, this variety complicates the process of estimating herd prevalence of infection as different sample types can have different values of sensitivity and specificity [10]; furthermore, a sample type's sensitivity and specificity can change over time because of the infection dynamics of PCV3 within a herd [11]. One key area that has to be taken into account when determining the optimal sampling procedure to estimate herd PCV3 prevalence is the use of individual vs. pooled samples. Individual samples can be useful for estimating animal level prevalence within a farm, but pooled samples can be useful for determining herd status as they may increase the odds of detecting the virus (and thus have greater sensitivity at the herd level). This issue was highlighted by our previous research on the effect of sample type on detection rates of PCV3 [10]. We identified increased detection rates in pooled samples (udder skin wipes [UW], placental umbilical cord [PUC] blood, and processing fluid [PF]) compared to individual samples (colostrum) and also showed that detection rates for UW remained stable over time prior to weaning, even though viral load decreased significantly over time.

However, those findings need further confirmation as the interpretation of our results may not be as simple as presented. In this study, along with PF, blood from the PUC, we collected additional piglet samples, that is, oropharyngeal swab (OS) and oral fluid (OF). Both of the additional sample types are useful to assess the infection in piglets during the pre-weaning period. OS samples collected from individual piglets provide information on viral shedding at the individual level; whereas OF are pooled samples collected from rope chews and can therefore be useful for monitoring infection at the group level. Furthermore, we collected samples from sows (blood swabs and whole blood) to examine changes in PCV3 infection over time and to explore the relationship between PCV3 replication and changes in hormone concentrations, which may influence viral replication within the host [12].

The aims of this study were to (1) compare detection rates among samples from piglets in the farrowing room, that is PF, PUC, OS, and OF; (2) to use OS from nursing piglets to explore the changes in PCV3 prevalence in the pre-weaning period; (3) to investigate the changes in PCV3 detection rates in sows before and after parturition and explore whether these differences are related to changes in oestrogen concentrations.

## 2. Materials and Methods

### 2.1. Farm Selection

The study was conducted on a large-scale pig farm in Shandong, China, which was an integrated breeding facility with three production lines. Each production line maintained a herd size of 8000 sows, utilising a continuous production model with piglets weaned on a weekly at ~21 days of age. Prior to the study, the farm was confirmed as being negative for porcine reproductive and respiratory syndrome virus (PRRSV) and porcine epidemic diarrhoea virus (PEDV), but the previous investigation had suggested the presence of PCV2, PCV3, rotavirus, and influenza A virus. Just prior to the study, the farm reported a sow abortion rate >5%, higher than its baseline rate of 2%–3%; however, there had been no reported increase in stillborn or mummified fetuses. Due to the biosecurity concern, only one production line was selected for the sampling collection.

### 2.2. Sample Collection

Pigs were sampled with the approval of the farm owner and adherence to the farm's biosecurity protocols, ensuring no disruption to farm production. All procedures were approved by the Institutional Animal Care and Use Committee of Nanjing Agricultural University (NJAULLSC2022031).

From July to August 2022, two trained technicians collected samples from all sows and their litters. The litter samples included PUC, PF, OS, and OF. For PUC and PF, the sample collection procedure was as per Wang et al. [10]. In brief, PUC was collected from three piglets per litter as soon as possible after birth and then pooled. PF was collected after castration (at ~3–5 days of age), with all testes from a litter being placed into a sterile container, 5 mL of saline added, and the contents mixed. About 1 mL of the mixed fluid was transferred into a sterile centrifuge tube.

OS were collected within 24 h of farrowing from all piglets in a litter. The technicians restrained the piglets with their heads immobilised and subsequently swabbed the soft palate and the left and right surfaces of the oropharyngeal area, three times per site, with a sterile cotton swab. After collection, the cotton swab was deposited into a sterile centrifuge tube containing 1 mL of saline. Additional OS samples were collected from two to three identified piglets per litter on days 1, 7, 14, and 21 after birth.

On day 21, OF samples were collected from piglets using cotton ropes provided for chewing. These ropes were placed in areas accessible solely to the piglets, with three unravelled strands hanging ~3 cm from the floor for at least 30 min. After 30 min, OF was collected by grasping the rope and squeezing it through gloved, clenched fingers into a sterile OF collection bag (Vetcome, Nanjing, China) [13]. Subsequently, 1 mL of the OF was transferred into a 2 mL centrifuge tube using a disposable sterile pipette. Once collected, the PUC, PF, OS, and OF samples were all refrigerated on the farm and transported on ice to the Immunology Research Institute of Nanjing Agricultural University within 24 h of collection. There, these samples were stored at −20°C until further testing (all samples were tested within 7 days of arrival at the laboratory).

Blood swab samples were collected from sows before and after farrowing. These samples were obtained using a needle prick at the base of the sow's tail. The skin at the tail base was wiped with sterile gauze soaked in saline and then pierced with a sterile needle. A sterile cotton swab was then placed at that site to collect the exuded blood. The cotton swab was then inserted into a sterile centrifuge tube containing 1 mL of saline. Blood swab sampling was undertaken at two different time periods: (1) July–August 2022 (contemporaneous with the piglet sampling; *n* = 51 sows) and (2) October 2022 (unrelated to any piglet sampling; *n* = 84 sows). At both time periods, three samples were collected per sow: (1) 3 days prior to the expected date of farrowing, (2) 3 days after farrowing, and (3) 8 days after farrowing.

Additionally, in October 2022, 5 mL of blood was collected at the same time as the blood swab samples were collected from 34 sows (convenience selection). Blood was collected from the external jugular vein into blood collection tubes without anti-coagulant (HUAEN, Nanjing, China) and then refrigerated in the farm's refrigerator before being transported within 24 h on ice to the Immunology Research Institute of Nanjing Agricultural University. There, the blood was centrifugated at 845 g for 10 min, with 1.5 mL serum being collected and placed into 2 mL cryotubes. All samples were then placed into a −80°C freezer and stored until further testing.

### 2.3. DNA Extraction and qPCR Test

All samples were tested separately, and laboratory staff were blinded to which animals the samples came from and, therefore, were not able to compare results across a sow/litter. DNA extraction was performed using the RM201-02 Virus DNA/RNA Purification Kit (Vazyme, Nanjing, China) with the VNP-32P automatic nucleic acid extractor (Vazyme, Nanjing, China), following the manufacturer's instructions. PCV3 detection was carried out using qPCR as per Franzo et al. [14]. In brief, 2 µL of extracted DNA was added to a PCR mixture containing 10 µL 2 × Premix Ex Taq (TAKARA Bio, Japan), 0.4 µL 50 × ROX Reference Dye (TAKARA Bio, Japan), and 0.6 and 0.3 µM of PCV-3–specific primers and probe, respectively. Double-distilled water was added to bring the final volume up to 20 μL. The cycling conditions were 95°C for 7 min, followed by 45 cycles at 95°C for 10 s and 60°C for 30 s. Assay efficiency was determined using the standard plasmid pCC1-4k-PCV3, which contains the entire PCV3 genome synthesised from a PCV3 sequence (accession no: KT869077). The plasmid was serially 10-fold diluted (10–10^8^ copies/μL) prior to the determination of assay efficiency to establish a standard curve.

### 2.4. Measurement of Plasma 17*β*-Oestradiol Concentration

17*β*-Oestradiol concentration (oestrogen) was measured using a commercially available ELISA kit (R&D systems, Bio-Techne, Minneapolis, USA) according to the manufacturer's instructions. Assay sensitivity was 12.1 pg/mL 17*β*-oestradiol.

### 2.5. Statistical Analysis

All data were visualised and analysed using R, version 4.3.0 [15], except where stated. The proportions of PCV3 positive results from different samples using the qPCR were computed with 95% confidence interval (95% CI) being constructed using the likelihood ratio method. The difference in detection rates was then compared using a univariable mixed-effects logistic regression model to account for potential clustering effects within individuals. The regression coefficients were converted as per Yang et al. [16] to represent a “population average” interpretation. All parameters, including the three regression coefficients (i.e., the intercept [logit of the proportion of positive detected by the PUC] and the effects of OS and OF, respectively, and the variance of the random effect *σ*_*A*_^2^ presenting the within-animal variability) were estimated using the Markov chain Monte Carlo (MCMC) method. A diffuse prior *N* (0, 0.001) was specified for all regression coefficients, where 0.001 is the precision parameter (the reciprocal of a variance in a standard parameterisation of a normal random variable), while a *Γ* (1,1) prior was used for the reciprocal of *σ*_*A*_^2^. The model was constructed using OpenBUGS [17], with one MCMC chain running for 50,000 iterations after discarding the first 1000 as a burn-in period to ensure convergence (assessed by the trace plots). Sensitivity analysis was conducted by using a different prior for *σ*_*A*_^2^, with a Unif (0,1) being specified for *σ*_*A*_. Gwet's AC1 [18] was used to assess the agreement between each of the six pairs of the sample types, with the Landis & Koch benchmarking scale [19] being used to interpret the level of agreement based on cumulative membership probability rather than point estimates.

The effect of time on the detection rate of PCV3 in OS samples and blood swab samples was analysed using a univariable generalized estimating equations (GEE) approach with a binomial family, logit link function, and a compound symmetry correlation structure. To study the association between the changes in PCV3 detection rate and oestrogen concentrations in sows, we built two competing causal diagrams to represent two different causal pathways. A multivariable logistic regression via GEE, including both time and oestrogen as independent variables, was built to test both hypotheses presented in the causal diagram. The interaction between time and oestrogen concentration was then tested based on the Wald test.

## 3. Results

### 3.1. PCV3 Detection in PUC, Testicular PF, OS, and OF Samples

**Figure 1:**
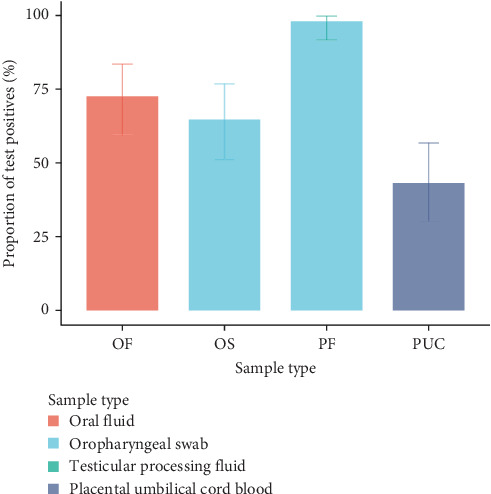
Detection of porcine circovirus type 3 by qPCR across four different sample types collected and aggregated at litter level.

PCV3 was detected in all sample types. The proportions of positive samples for each of the four piglet sample types are shown in Figure 1. The highest detection rate was observed in the PF samples (50/51; 98%). Data from PF samples was excluded from the subsequent modelling process because of this imbalanced distribution.  Table 1 summarises the effect of the remaining piglet sample types on the PCV3 detection rate. Both OS and OF samples had higher detection rates for PCV3 than PUC. However, our results did not separate OF from OS on detection rate (odds ratio [OR] of detection for OS vs. OF was 0.69, 95% credible interval [95% CrI]: 0.31–1.52). All results were insensitive to the different priors for *σ*_*A*_^2^.

Table 2 shows the agreement between the three sample types. For OS vs. OF, the point estimate of Gwet's AC1 was 0.38 (95% CI 0.1–0.66), representing fair agreement; however, the probability that the agreement was at least fair (~0.9) was not >0.95. The results for PUC samples strongly suggest that there is, at best, limited agreement with those from OS/OF.

### 3.2. PCV3 Detection Rates in OS Sample of Nursing Piglets

**Figure 2:**
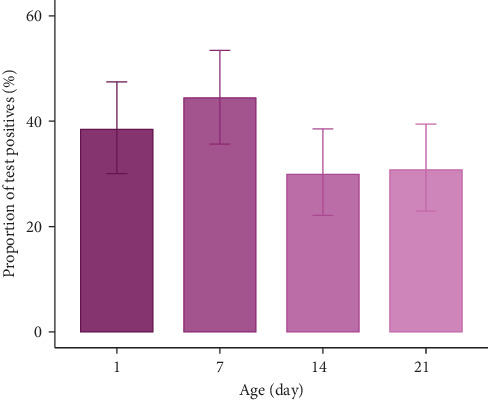
Detection of porcine circovirus type 3 using qPCR by oropharyngeal swab from piglets among different age groups.

OS samples were collected from a total of 117 individual piglets from 51 litters on days 1, 7, 14, and 21.  Figure 2 illustrates the detection rate of PCV3 on those days. The detection rate ranged from 35/117 (30%; day 14) to 52/117 (44%; day 7) across the four time points (Table 3). Compared to samples taken on day 21, samples taken on day 1 and day 7 had moderately higher odds (OR 1.41 and 1.8, respectively) of detecting PCV3 (Table 3).

### 3.3. Absence of Causation Between Oestrogen and PCV3 Detection

**Figure 3:**
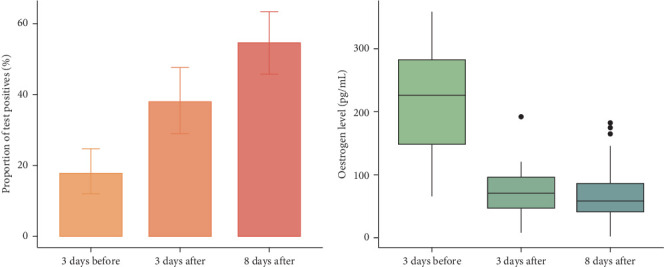
Time patterns of detection of porcine circovirus type 3 in needle prick blood samples and serum oestrogen concentrations in sows over the farrowing period.

Due to transportation issues during the first sampling period, sample collection was compromised; only 35 sows had blood swab samples collected 3 days before and 8 days after farrowing, while for the remaining 16 sows, samples were only taken 3 days before and 3 days after farrowing. Including the 84 samples collected in Oct 2022, this led to us having blood swab samples from a total of 135 sows, with 100 matched samples for 3 days before and after farrowing and 119 matched records for 3 days before and 8 days after farrowing. Over this period, the detection rate increased (Figure 3A). Compared to 3 days before farrowing, the odds of detecting PCV3 virus were 2.86 (95% CI: 2.3–3.56) times higher 3 days after farrowing, and compared to 3 days before farrowing, samples taken 8 days after farrowing had higher odds of PCV3 detection (OR 6.37; 95% CI: 5.13–7.92).

**Figure 4:**
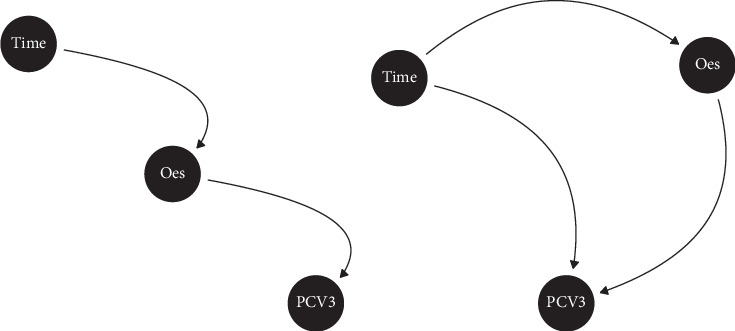
Two competing causal diagrams representing the hypothetical causal relationships between oestrogen concentration (17β-oestradiol) and the detection of porcine circovirus type 3 in sows over the farrowing period.

In contrast, oestrogen concentration was highest 8 days before farrowing (median = 226, first/third quartiles: 148–282) and markedly lower after farrowing, with median = 70.8 (46.9–96.3) and median = 58.3 (41.1–86.1) 3 and 8 days post-farrowing, respectively (Figure 3B). Two competing causal diagrams are presented to explain the observed association (Figure 4). Figure 4A suggests that the oestrogen concentration changes with time after farrowing and that this change (potentially) causes PCV3 to become more likely to be detected. In contrast, Figure 4B treats the “time” variable as a confounder. According to the multivariable logistic regression model, the apparent negative association between oestrogen and PCV3 detection was, however, not a causal relationship. After controlling for oestrogen concentration, the presence of a statistically significant time effect precluded the validity of the causal hypothesis summarised in Figure 4A. After adjusting for this time effect, no clear association was observed between oestrogen concentrations and PCV3 detection rate (*p*-value = 0.13). Thus, across the 34 sows tested for PCV3 and 17*β*-oestradiol, we were not able to show a relationship between oestrogen concentration and PCV3 detection.

## 4. Discussion

In this study, we compared four different sample types for PCV3 detection in piglets and detected the virus in all four sample types. However, none of the sample type pairs showed sufficient agreement for them to be used interchangeably. As in our previous study [10], the sample type with the highest detection rate of PCV3 in this study population was PF (50/51 samples). However, the detection rate in PF samples in the current study was much higher than the 71.6% (95% CI: 63.2%–79.1%) we reported in Wang et al. [10]. It is unclear why the detection rate was so much higher in the current population than in our previous study. Nevertheless, our finding that the detection rate associated with PF was higher than that of PUC is consistent with both Wang et al. [10] and Igriczi et al. [20]. The higher rate of detection for PF vs. PUC may be related to different sampling time points. PUC samples are collected immediately after birth, while PF samples are collected at ~3–5 days of age. Hence, PUC samples are more likely to reflect the disease status of newborn piglets, that is, they reflect infection during gestation [21], while PF samples reflect infection during gestation and also shortly after farrowing [22]. Though data from PF samples were not included in our statistical modelling, the high rate of detection in this and our previous study [10] suggest that PF is likely to be a suitable sample type for PCV3 testing in piglets if maximising sensitivity is of priority.

The OF and OS samples also showed higher detection rates than that of PUC samples. As with PF, these higher rates are likely related to horizontal transmission between piglets (thus probably reflecting a higher true prevalence rather than better detection). However, the difference may, at least in part, also be related to the variation in the number of piglets included in each pooled sample, with PUC samples being collected from only three piglets per litter, while OF and OS being collected from the entire litter. The detection rates of the OS and OF samples on day 21 were similar, but the limited agreement between the two sample types indicates that OS and OF samples identify different subsets of infected animals. Further studies are required to establish the reason for this limited agreement.

The analysis conducted in the pre-weaning piglets using OS samples suggests that PCV3 is most likely to transmit between piglets in the first week after farrowing and that after this timepoint, the proportion of piglets shedding reduced. This is consistent with our finding from UW that viral loads decrease over time [10]; thus, both OS and UW could potentially be useful to study the dynamics of PCV3 in piglets in the farrowing room. Nevertheless, we believe that OS samples are likely to be more useful than UW even though their collection is more challenging and may cause stress to piglets, because although UW are cheap and easy to collect, positive results from UW could occur as a result of contamination by material other than OFs, whereas OS samples preclude the possibility of such contamination. Furthermore, OS can be collected at an individual level and thus may be a more accurate method of monitoring PCV3 infection in the farrowing room. However, further investigation is required to confirm this conclusion and to identify whether sows acquire new infections in the farrowing room when newborn piglets begin shedding large amounts of the virus during the first week of life.

Oestrogen has a regulatory effect on the immune system, therefore, may affect viral replication within the host, with some viruses showing reduced replication in response to increased oestrogen concentrations (e.g., influenza A and human immunodeficiency virus) [23–25]. Furthermore, viruses may affect oestrogen concentrations; for example, in pigs, Wang [26] found the PRRSV-reduced oestrogen concentrations in late pregnancy. In this study, we found, as expected [27], that serum oestrogen concentrations decreased markedly between 3 days before and 3 days after parturition, with no significant change between 3 and 8 days after parturition (Figure 4). In contrast, the PCV3 detection rate increased markedly between 3 days before and 3 days after parturition and continued to do so between 3 days after and 8 days after parturition. However, our modelling suggested that this apparent relationship was confounded by time; that is, in the causal diagram (Figure 4B), this means that the direct path between “Oes” and “PCV3′ does not exist, indicating the absence of a causal effect of oestrogen concentrations on PCV3 detection. However, the relatively low number of sows included in this modelling (*n* = 34) means that this conclusion needs further testing in a larger number of sows across multiple farms.

## 5. Conclusions

Building on our previous study, this study compared the agreement across four sample types in piglets. Our findings add further evidence that PFs can be suitable samples to detect PCV3. In addition, we demonstrated that OSs and OFs could be used to monitor PCV3 at the group level, although they may not be suitable for identifying infections at the individual or litter level. The OSs have additional use that can help monitor PCV3 dynamics in the farrowing rooms during the pre-weaning period when the virus was constantly detected. Finally, we found no evidence of an association between PCV3 infection and oestrogen concentrations during the farrowing time. These findings help identify the best sample types for PCV3 detection on a sow farm; further investigations are required to estimate the sensitivities and specificities of the test strategies using the corresponding samples. This information is useful to understand the prevalence patterns of PCV3 at different production phases and develop effective control methods.

## Figures and Tables

**Table 1:** Effect of sample types, including blood from the placental umbilical cord (PUC), oropharyngeal swab (OS), and oral fluid (OF), on the proportion of porcine circovirus type 3 positives tested using a qPCR.

Sample type	Positives	Total	Odds ratio	95% Credible interval	
				Lower	Upper
PUC	22	51	Reference	—	—
OF	37	51	3.69	1.7	8.23
OS	33	51	2.51	1.19	5.48

**Table 2:** Agreement measured as Gwet's AC1 coefficients between sample types.

Benchmark	Magnitude	Cumulative membership probability		
		PUC vs. OF^a^	PUC vs. OS^b^	OF vs. OS^c^
(0.8, 1)	Almost perfect	0	0	0.001
(0.6, 0.8)	Substantial	0	0	0.056
(0.4, 0.6)	Moderate	0.007	0.018	0.443
(0.2, 0.4)	Fair	0.141	0.246	0.904
(0, 0.2)	Slight	0.617	0.769	0.997
(−1, 0)	Poor	1	1	1

Abbreviations: CI, confidence interval; OF, oral fluid; OS, oropharyngeal swab; PUC, placental umbilical cord.

^a^Gwet's AC1 = 0.043 (95% CI: −0.249–0.336).

^b^Gwet's AC1 = 0.104 (95% CI: −0.179–0.386).

^c^Gwet's AC1 = 0.380 (95% CI: 0.102–0.658).

**Table 3:** Age effect on the qPCR result of porcine circovirus type 3 detection using oropharyngeal swab from piglets.

Age (day)	Positives	Total	Odds ratio	95% Confidence interval	
				Lower	Upper
1	45	117	1.41	1.15	1.72
7	52	117	1.8	1.46	2.22
14	35	117	0.96	0.79	1.17
21	36	117	Reference	—	—

## Data Availability

The data that support the findings of this study are available from the corresponding author, Zhen Yang, upon reasonable request.
